# Design and Fabrication of a Novel Microfluidic System for Enrichment of Circulating Tumor Cells with the Assistance of Computer Simulations

**Published:** 2019

**Authors:** Dina Dorrigiv, Manouchehr Vossoughi, Iran Alemzadeh

**Affiliations:** Department of Chemical Engineering, Sharif University of Technology, Tehran, Iran

**Keywords:** Circulating tumor cells, Computer simulation, Microfluidics, Prognosis

## Abstract

**Background:**

Cancer is the first cause of death in developed countries. The heterogeneous nature of cancer requires patient-specified treatment plans. One reliable approach is collecting Circulating Tumour Cells (CTCs) and using them for prognosis and drug response assessment purposes. CTCs are rare and their separation from normal cell requires high-accuracy methods.

**Methods:**

A microfluidic cell capture device to separate CTCs from peripheral blood is presented in this study. The CTC separation device applies hydrodynamic forces to categorize cells according to their sizes. The proposed device is designed and evaluated by numerical simulations and validated experimentally. The simulation modified design was fabricated by soft lithography which allows prototyping the device in a few hours. For experimental setup two solutions: 1) fixed cells spiked in Phosphate Buffered Saline (PBS), and 2) fixed cells in blood were used. The CTC separation device was validated by tracking the flow and separation of cancer cell lines in the solutions.

**Results:**

It is demonstrated that the setup is capable of CTC enrichment up to 50 times.

**Conclusion:**

The presented CTC enrichment method reduces costs by eliminating the use of antibodies. The high-throughput method has the potential to be used in preclinical studies of cancer.

## Introduction

According to World Health Organization (WHO), cancer is one of the first common death causes worldwide. Governmental reports of highly developed countries such as Canada and United Kingdom show that one of two people will develop cancer during their lifetime 1. Cancer in early stages is curable. Metastasis, the process in which cancer spreads throughout the body, usually starts at stage three of the cancer. As metastasis happens, cancer becomes hard to control and makes way to different organs 2. Prognosis demands patient samples to trace the cancer. The commonly used sampling method is biopsy which is sampling the tumor tissue through surgery. Biopsy has several drawbacks. The idea of replacing biopsy with a minimally invasive approach has been the subject of several studies over the last decades. Liquid biopsy which mainly refers to blood draw is a conventional and widely used test to observe the tumor biomarkers. Blood draw may not carry all the tumor characteristics in early stages of cancer. However, in patients dealing with metastasis, blood becomes a valuable source of information. By the start of metastasis, tumor cells start circulating in the blood. The Circulating Tumor Cells (CTCs) fully represent the characteristics of the tumor. They account for about 0.004% of all nucleated blood cells beside the tumor site, and they are less in distant parts from the tumor. CTCs' short lifetime makes their enrichment more challenging 3,4. Separation and enumeration of CTCs provide the clinicians with patient-specified information. If isolated alive, CTCs can be cultured and used in Therapeutic Testing Assays (TTA). These predictive assays measure the response of CTCs to therapeutics by exposing the cells to different chemotherapeutic agents and evaluating their response 5,6.

CTC isolation has been a hot topic in cancer engineering in recent years and several approaches are proposed. CTCs’ unique biochemical and physical characteristics make them identifiable from other blood cells. Biochemical-based separation methods target cells’ biochemical properties such as surface proteins to mark and isolate them from blood cells. In this approach, cells are incubated with certain types of antibodies which bind to their surface antigens and bring about further separation. According to the available antibodies, separation could be negative or positive. In negative separation, antibodies target non-cancerous blood cells (*e.g*., targeting leukocytes with anti-CD45), and in positive separation, CTCs are the target (*e.g*., targeting HeLa cells with anti-EpCAM) 7,8. Biochemical based separation has been adopted by several researchers. However, by the advent of Cancer Stem Cells (CSC), this method seems untrustworthy. CSCs are tumor cells that are able to differentiate into different phenotypes and form new tumors as the disease progresses. They resemble healthy stem cells in having similar surface proteins which make biochemical based separation tricky 3.

Physical-based approach focuses on physical properties of cells (*i.e*., size, density, electrical permittivity, dielectric characteristics, and adhesiveness among cells) to isolate them. Considering that CTCs are mostly in the range of 15 to 30 *μm* in diameter, while the diameters of red and white blood cells are mostly below 15 *μm*, size-based separation seems a promising approach 7,9.

CTC separation methods including biochemical or physical ones are either active or passive according to the tools they exploit to separate the cells. Active methods impose an external force field (*i.e*. electrical, magnetic or acoustic force field) on the cells to separate them. Passive methods separate the cells by manipulating hydrodynamic forces of a laminar flow in a channel 10. Passive methods require less equipment and are simpler to assemble in comparison to active methods. The use of inertial microfluidics for a physical-based, passive cell capture device was introduced by Dicarlo in 2007 11. While most of the microfluidics devices work with fully creeping (stokes) flow regimes (*i.e*., Re<<1), inertial microfluidics has moved the boundaries and works in laminar and transient flow regimes with 1<Re<100. Devices that are designed to work with inertial microfluidics have simple structures and allow higher flow rates to pass in a shorter time scale compared to other types of pf microfluidic devices. Straight, spiral, serpentine and multi-orifice are different structures of inertial microfluidic separation systems 12–16.

In this paper, an inertial microfluidic CTC separation device was introduced. Our device is a straight channel with six contraction and expansion arrays. Each contraction array is separated from the next one by a semicircular expansion array.

### Theoretical background

According to Sergé and Silberberg’s observations, in an annular cross-section channel, particles moving in a laminar flow find equilibrium positions at a distance of about 0.6 times the radius from the center of the tube 17. Particles are subject to viscous drag forces in the same direction of flow streamlines, and to inertial lift forces perpendicular to the flow streamlines. Several empirical studies have shown that particles find different equilibrium positions on the plane perpendicular to their moving direction. The equilibrium position varies by varying the size of particles 18.

Inertia is responsible for the lateral migration of particles in a tube. Inertial lift forces in a Poiseuille flow consist of two countercurrent forces: 1) Shear-induced lift force due to the parabolic nature of velocity profile; the direction of this force is from the center toward the walls of the channel. Shear-induced lift force pushes the particles moving near the center of the channel to the walls, 2) Wall-induced lift force which is created by the pressure gradient between the particles and the walls, and pushes the particles near the wall to the center 19. The amount of inertial lift force on particles is calculated by Equation 1 20.

Equation 1:

$$F_{L}=f_{L}\rho_{f}U^{2}\frac{a^{4}}{H^{2}}$$

Where fL is the lift coefficient, a function of particle’s position and channel’s Reynolds number, ρf and U are density and velocity of the fluid respectively, a is the particle’s diameter, and H is the hydraulic diameter, the diameter of the cross-section. The hydraulic diameter is used to handle the flow calculations in a non-circular channel, the same as a round channel. In a rectangular cross-sectioned microchannel, Equation 2 calculates hydraulic diameter where w and h are width and height of cross-section.

Equation 2:

$$\text{H}=\frac{2\text{wh}}{\text{w}+\text{h}}$$

For particles moving with a laminar flow in a straight channel, the dominant forces are inertial lift force and viscous drag force. The effect of other forces should be taken into account under certain circumstances. One of the factors that can alter the forces on particles in a micro channel is geometry. W.R. Dean's mathematical solution along with empirical observations has shown that particles in laminar flow change their movement direction after entering a curvature due to the centripetal forces. This change in the direction of motion causes a pressure gradient between the center and walls of the channel that superposes a secondary flow on the flow and creates two countercurrent vortices from the center toward the walls and backward. These vortices are called Dean vortices after the name of the theoretician. Dean vortices drag the particles which gives rise to the formation of Dean drag force. The direction of Dean drag force is perpendicular to the flow streamlines^18^,^20^,^21^.

The magnitude of the Dean drag force is calculated by Equation 3, Where U_Dean_, refers to the velocity of Dean vortices and μ is the viscosity of the fluid.

Equation 3:

$$F_{D}=3\pi \mu U_{\text{Dean}}a$$

Dean drag force in combination with inertial lift force disturbs particles from their streamlines according to their sizes 20. Particular geometries mimic the effect of curvature in straight channels. Recently published studies of Park *et al* has shown that adding sudden contractions in a straight channel can also cause the formation of the Dean Drag forces. Secondary flows which are the same as Dean vortices are created in the entrance of contraction region, where the flow is accelerated 15, 16,22–24. Our CTC separation device is a straight channel with contraction arrays that separates the cells based on their sizes.

## Materials and Methods

### COMSOL simulation

**Figure 1.**
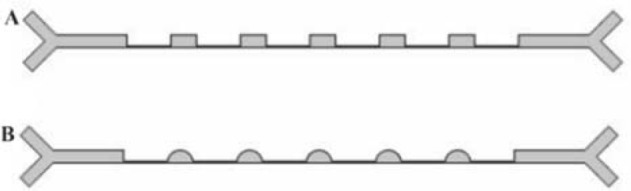
A: The CEA and B: the smCEA drawn in COMSOL multiphysic.

There are several empirical studies on inertial micro-fluidic separation devices. However, to our knowledge, no group has focused on the computational simulation of these devices. In this study, simulation was conducted based on the data from a past empirical work called the Contraction Expansion Array (CEA) as a reference (Figure 1A) to verify the simulations 16. The CEA is a straight channel with six contraction regions, two inlets, and two outlets. The dimensions of the CEA are in table 1. The general objective in this part is to modify the configuration of the system to improve the separation efficiency. From theory, it is known that both Dean Drag force and inertial lift force are affected by the geometry of the channel. Hence, manipulating the channel geometry can affect the separation. The design of the CEA was elaborated by manipulating its geometry with a software. The software COMSOL Multiphysics 5.2® was used to simulate the 3D module of the particles flowing in the CEA. Primary assumptions were about rigid spherical particles diffused in a laminar flow of a liquid with the characteristics of water. Laminar flow and particle tracing physics are the physics used in COMSOL environment. Dean drag force equation is assumed to follow stokes drag equation all over the device and the inertial lift force term was inserted manually, different for contractions and expansions regions. Then, the effect of the angle between two inlets, the depth of the channel, the shape of expansion arrays and the ratio between the two inlet flow rates with simulation were evaluated. Figure 1B shows the properties of the optimized CEA according to simulation results. The optimized device is called the simulation-modified CEA (smCEA).

**Table 1.** Dimensions of the CEA

**Parameter**	**Sign**	**Initial amount**	**Unit**
**Depth**	H	63	*μm*
**Width of expansion**	W_E_	50	*μm*
**Width of contraction region**	W_C_	350	*μm*
**Length of the contraction region**	L_C_	1200	*μm*
**Length of the expansion region**	L_E_	700	*μm*
**Number of contraction region**	N_C_	6	-
**Degree between two inlet valves**	θ	100	deg
**Particle-fluid flowrate**	Q_P_	0.3	*ml/hr*
**Focusing-fluid flowrate**	Q_F_	6	*ml/hr*
**Ratio of particle-fluid to focusing-fluid**	η=Q_F_/Q_P_	20	-

### Fabrication of the design

**Figure 2.**
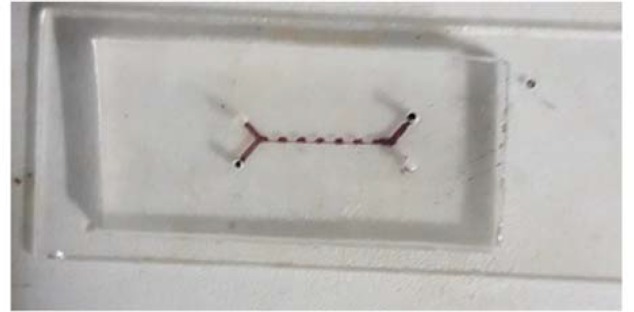
Fabricated device prepared with soft lithography.

The smCEA was fabricated according to the simulation outcome. The smCEA, similar to the CEA, has two inlets for a flow containing cells (particle fluid) and a particle-less fluid with the same physical characteristics (focusing fluid) to focus on the particles, and two outlets. The devices were prepared by soft lithography protocol 25. In this method, molds of the smCEA were prepared with SU-8 photoresist on a glass substrate following the conventional photolithography process. Then, Poly Di-methyl Siloxane (PDMS) and its curing agent (Sylgard 184; Dow Corning, MI) in a ratio of 10:1 were poured on stamps and cured in a 65° oven overnight. The PDMS layer was detached from the stamp and bonded to a glass slide by treating both surfaces with an air-plasma treatment process in low pressure (500 mTorr) chamber (Figure 2). The plasma treatment process made PDMS hydrophilic.

### Sample preparation

Two approaches in the preparation of the particle fluid were used while the focusing fluid was the same during the experiments. PBS was used as the focusing fluid. For the first set of experiment, a combination of fixed mouse fibroblast cell line (L929) (10–15 *μm* in diameter) and breast cancer cell line (MCF7) (18–25 *μm* in diameter) with a predefined density was suspended in PBS to form the particle fluid. For the second set of experiment, first, the whole blood was diluted 100 times with PBS, and then a suspension of MCF7 fixed cells was spiked into the blood. Whole blood was obtained from Iranian Blood Transfusion Organization (IBTO). MCF7 fixed cells that represent CTCs were stained with DAPI (Sigma Aldrich; D9542) before the experiments to facilitate further visualization.

### Experimental setup

Before running the system, to sterilize the device, ethanol was injected in the device and heated in a convection oven at 180 *°C* for 2 *hr*. Then, sterilized PBS was passed through the device to eliminate air bubbles. The particle-fluid and PBS were injected into the channels by a syringe pump. Two syringe barrels with a 1:10 diameter ratio containing particle-fluid and focusing-fluid, respectively were installed in a single syringe pump (SP1000, FNP co.) to inject the fluids with the same speed, resulting in a 1:10 flow rate ratio.

**Figure 3.**
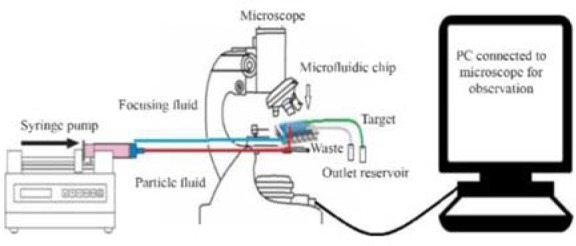
Schematic design of the experimental setup.

Two reservoirs, open to the atmosphere, were connected to the outlets of the device to collect the samples that leave the channel. The motion of particles in the channels were tracked by a microscope. Figure 3 illustrates the experimental setup.

### Cell observation

**Figure 4.**
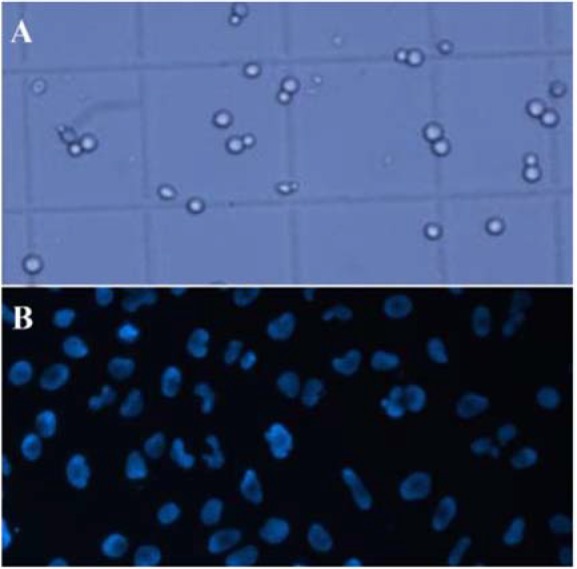
Cells on a Neubauer chamber under the microscope, A) all the cells, B) DAPI stained cells under the fluorescent filter.

MCF7 cells are the target cells to be separated. The separation efficiency is dependent on the difference of MCF-7 cells in the inlet and outlet. Initial density of MCF-7 in the suspension was known, and the number of MCF-7 cells could be counted in the outlets with a Neubauer chamber under the fluorescent filter of microscope (Figure 4).

## Results

This part is divided into two parts to discuss the results of computer simulation and the experimental observations.

### Simulation

The dimensions of the CEA were used, and each time, one of the measurements was changed while keeping the other parameters constant. The effect of the angle between the two inlets (θ), the depth of microchannels (D), the shape of contraction arrays and the ratio of focusing-fluid flowrate to particle-fluid flowrate (η) were evaluated through simulation. In the end, simulation was done for the CEA and the smCEA to compare their separation time and efficiency. The separation yield was 100% for both of the designs. Total separation time is shorter in smCEA compared to CEA.

### Focusing-fluid to particle-fluid flow rate (η)

**Figure 5.**
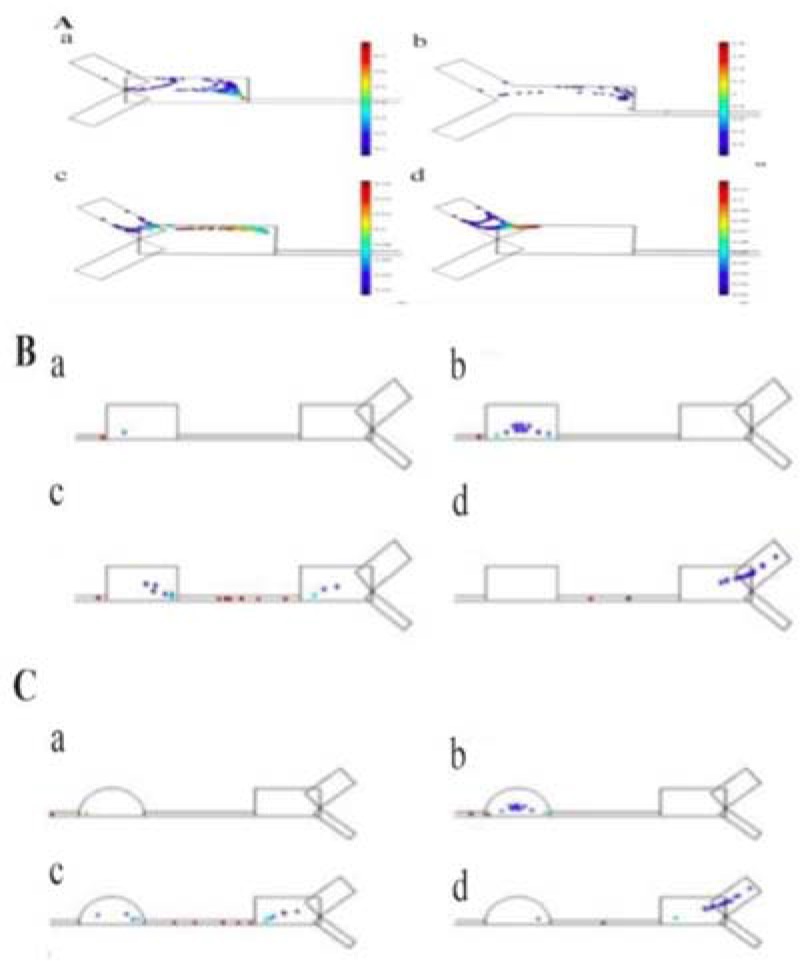
Effect of ŋ on particles arrangement, A) at the entrance and B) near the outlets of reference. C) near the outlets of the modified design, A) ŋ=1 B) ŋ=5 C) ŋ=10 D) ŋ=15 at 0.0015 s

Choosing an optimum η enhances particle arrangement and decreases the arrangement time. η in the CEA is 20. It was assumed that the flowrate of the particle-fluid was constant, and the flowrate of the focusing fluid was manipulated. By simulation, η was changed from 1 to 20 by a step of 5. In η=1, particles were disorganized. Increasing η from 1 to 15 aligned the particles. For η>15, a flow back happened to the particle-fluid and particles entered the focusing-fluid valve. The optimum ratio was 11 according to simulation results. Figure 5 shows the simulation results for different amounts of η.

### The angle between inlets (θ)

**Figure 6.**
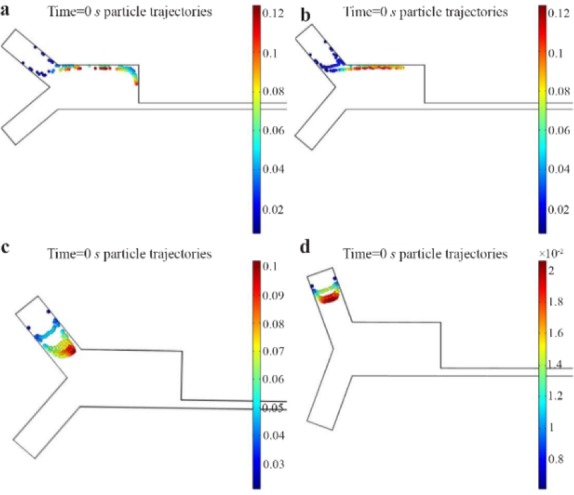
Effect of θ on particle arrangement a) θ=80° b) θ=100° c) θ=120° d) θ=140° at t=0.025 s.

The effect of the θ on particle alignment was similar to the η. The task of the focusing fluid was to focus the particles. With simulation, it was observed that the angle θ has a direct effect on time and quality of particle queuing. The θ was evaluated by changing it from 80 to 140 with a step of 20°. Figure 6 shows the effect of θ on particles’ alignment after the same elapsed time. Results show that particle arrangement improves and the alignment time decreases by increasing θ from 80° to 100°. By increasing θ above 100° particles align with delay. In θ>120° particles flow back to the inlets. According to simulation, the optimum amount is θ=100°, the same as the CEA.

### Depth of microchannel (D)

**Figure 7.**
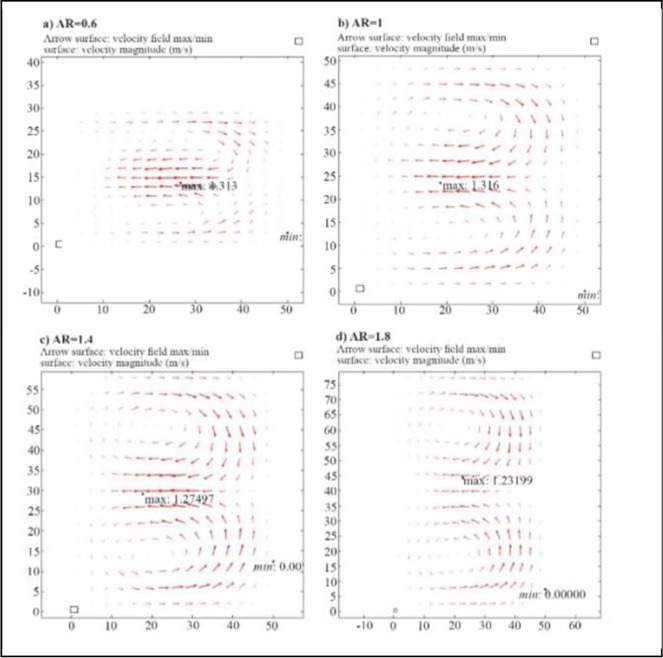
The effect of depth of channel (D) on Dean vortices. a) D=30 *μm* b) D=50 *μm* c) D=70 *μm* d) D=90 *μm*. AR refers to the ratio of the depth to the width of channel. Here the width is constantly 50 *μm*.

The depth of the channel (D) affects the formation of the Dean vortices and the magnitude of U_Dean_. D is increased from 30 *μm* to 90 *μm* with a step of 20 *μm*, while D=63 *μm* for the CEA. As shown in figure 7, Dean vortices are formed for D=30 *μm* to D=70 *μm*, and for depths more than 70 *μm*, chaos in the flow disturbs the vortices. The velocity of Dean vortices decreases by increasing the aspect ratio. According to the simulation results, the optimum depth is 50 *μm* to form the Dean vortices with the highest velocity.

### Shape of expansion arrays

**Figure 8.**
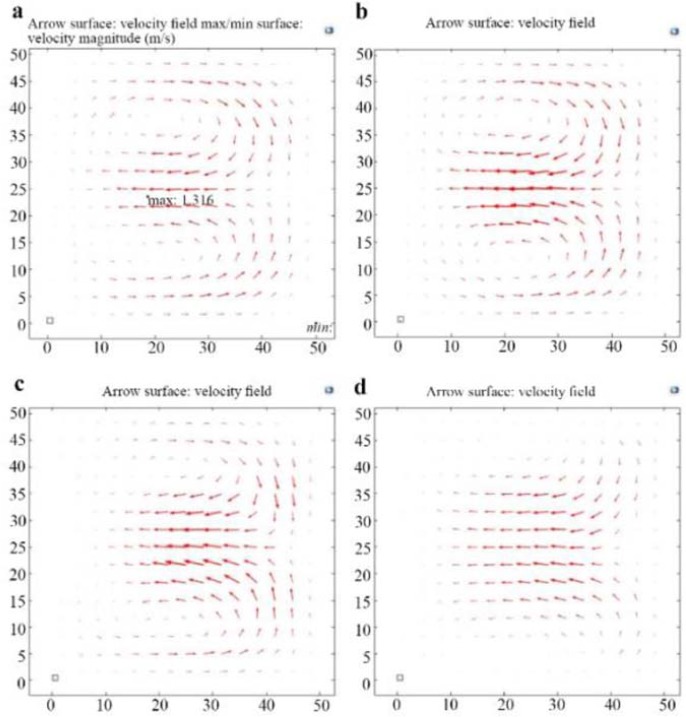
Effect of shape of expansion arrays on Dean vortices, a) rectangular arrays b) semicircular arrays c) triangular arrays and d) trapezoidal arrays.

The shape of expansion arrays is also effective on the formation of Dean vortices. A rounded structure is preferred to prevent trapping of the cells in the corners. Rectangle (as in the CEA), semicircle, triangle, and trapezoid where used for expansion arrays. As shown in figure 8, trapezoid shape prevents the formation of the Dean vortices. According to simulation results, triangular-shaped arrays result in high acceleration and chaos in the motion of particles. The acceleration creates a turbulence which may decrease the separation efficiency. Rectangular and semicircular expansion arrays show the best results according to simulation results. Table 2 shows the dimensions of the simulation modified CEA according to simulation results.

**Table 2.** Dimensions of the smCEA

**Parameter**	**Sign**	**Optimized amount**	**Unit**
**Depth**	H	50	*μm*
**Width of expansion**	W_C_	50	*μm*
**Width of contraction region**	W_E_	350	*μm*
**Length of the contraction region**	L_C_	1200	*μm*
**Length of the contraction region**	L_E_	700	*μm*
**Number of contraction region**	N_C_	6	*-*
**Degree between two inlet valves**	θ	100	deg
**Particle-fluid flowrate**	Q_P_	0.3	*ml/hr*
**Focusing-fluid flowrate**	Q_F_	3.3	*ml/hr*
**Ratio of particle-fluid to focusing-fluid**	η=Q_F_/Q_P_	11	*-*

### Experiments

**Figure 9.**
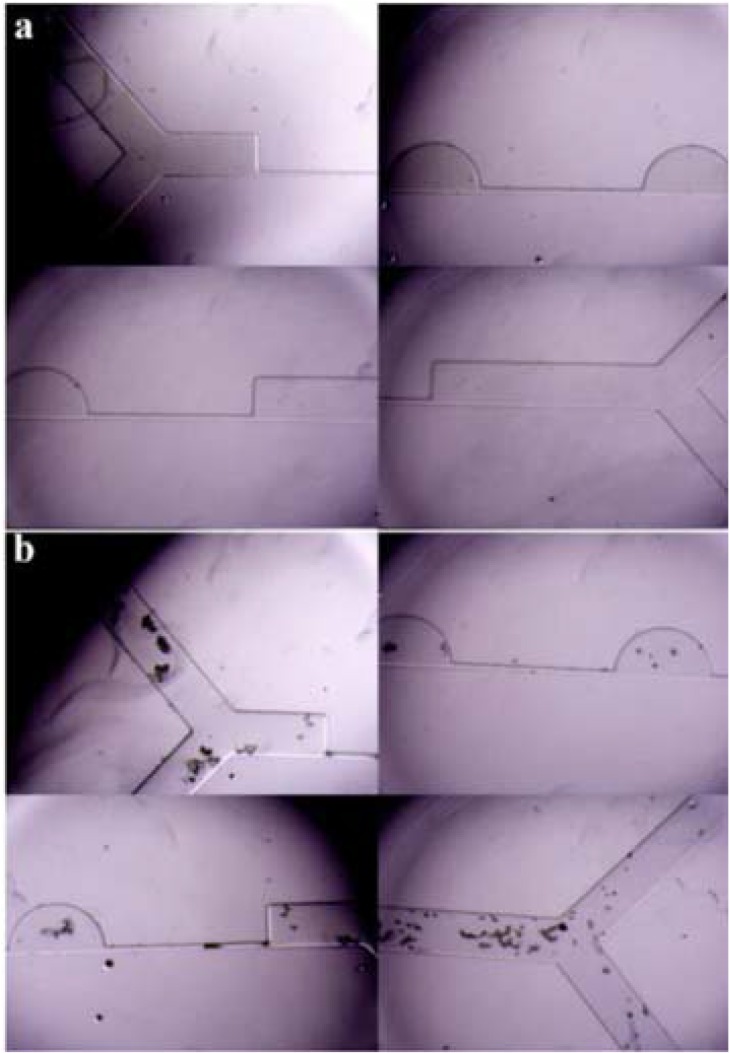
Microscope images from a) the first, and b) the second setup of the experiment.

Two sets of experiments were done for the CEA and the smCEA simultaneously. First, a mixture of L929 and MCF7 cells was injected into both systems and the outlet samples were collected to count the MCF7 cells. During their motion, fixed cells did not adhere to each other and moved freely (Figure 9A). Both systems can do the separation without clogging the channel during their ope ration. The first set of experiments shows a slight difference between the separation yield of the CEA and the smCEA.

In the second set of experiment, blood was diluted and the MCF7 cells were spiked into it. Cells adhered to each other and to blood cells, and clogged the channels after a short time (Figure 9B). This happens because diluted whole blood was used. Out of the veins, platelets clump together and form clots. Red blood cells lyse and coagulate in the flow. Red blood cell and platelets form clusters that trap fixed cells and clog the channels. The setup that works with blood shows higher separation yield for the smCEA than the CEA. However, the separation yield is less than the first set of experiments in both cases. One reason may be the short run-time of the setup. Tables 3 and 4 present the outcomes of the experiments.

**Table 3.** Experimental data of the first set of experiment

**Device**	**MCF7 to all cells in the particle fluid**	**MCF7 to all cells in the out-let**	**Operation time**
**The CEA**	37.5%	83.5%	Unlimited
**The smCEA**	37.5%	82.6%	Unlimited

**Table 4.** Experimental data of the second set of experiment

**Device**	**MCF7 to all cells in the particle fluid**	**MCF7 to all cells in the outlet**	**Operation time**
**The CEA**	26.7%	39.2%	170s
**The smCEA**	26.7%	47%	210s

## Discussion

### Focusing-fluid to particle-fluid flowrate (*η*)

Choosing an optimum amount for η enhances particle arrangement and decreases the arrangement time. η in the CEA is 20.

### The angle between inlets (θ)

The effect of the θ on particle alignment was similar to the η. The goal of the focusing fluid was to focus the particles. With simulation, it was observed that the angle θ has a direct effect on time and quality of particle queuing.

### Depth of microchannel (D)

The depth of the channel (D) affects the formation of the Dean vortices and the magnitude of U_Dean_. Increasing the aspect ratio decreases the velocity of Dean vortices.

### Shape of expansion arrays

The shape of expansion arrays is also effective on the formation of Dean vortices. A rounded structure is preferred to prevent trapping of the cells in the corners. Rectangle (as in the CEA), semicircle, triangle, and trapezoid where used for expansion arrays.

## Conclusion

The current research intended to conduct computer simulation for an inertial microfluidic CTC separation system and evaluate simulation besides experiments. Previous works mainly focused on empirical studies. A previously published system was chosen for separation of CTCs, called CEA and modified with the assistance of computer simulation by COMSOL multiphysics. The microfluidic devices were prepared with soft lithography process in a few hours. In the end, simultaneous experiments were conducted with the CEA and smCEA to validate the simulations. Experiments show higher separation yield for the smCEA. Simulation can save time and money by reducing the number of trial-error experiments. Microfluidic technology, assisted by computer simulation, helps to make trustworthy, inexpensive clinical tools. Future studies are necessary to focus on higher concentration of blood and patient-derived blood samples. All the experiments were done with fixed cells while clinicians deal with live cells. Experiments with live cells are necessary.
